# Toward Reflective Spiking Neural Networks Exploiting Memristive Devices

**DOI:** 10.3389/fncom.2022.859874

**Published:** 2022-06-16

**Authors:** Valeri A. Makarov, Sergey A. Lobov, Sergey Shchanikov, Alexey Mikhaylov, Viktor B. Kazantsev

**Affiliations:** ^1^Instituto de Matemática Interdisciplinar, Universidad Complutense de Madrid, Madrid, Spain; ^2^Department of Neurotechnologies, Research Institute of Physics and Technology, Laboratory of Stochastic Multistable Systems, Lobachevsky State University of Nizhny Novgorod, Nizhny Novgorod, Russia; ^3^Neuroscience and Cognitive Technology Laboratory, Center for Technologies in Robotics and Mechatronics Components, Innopolis University, Innopolis, Russia; ^4^Center For Neurotechnology and Machine Learning, Immanuel Kant Baltic Federal University, Kaliningrad, Russia; ^5^Department of Information Technologies, Vladimir State University, Vladimir, Russia

**Keywords:** spiking neural networks (SNNs), memristors and memristive systems, high-dimensional brain, plasticity, reflective systems

## Abstract

The design of modern convolutional artificial neural networks (ANNs) composed of formal neurons copies the architecture of the visual cortex. Signals proceed through a hierarchy, where receptive fields become increasingly more complex and coding sparse. Nowadays, ANNs outperform humans in controlled pattern recognition tasks yet remain far behind in cognition. In part, it happens due to limited knowledge about the higher echelons of the brain hierarchy, where neurons actively generate predictions about what will happen next, i.e., the information processing jumps from reflex to reflection. In this study, we forecast that spiking neural networks (SNNs) can achieve the next qualitative leap. Reflective SNNs may take advantage of their intrinsic dynamics and mimic complex, not reflex-based, brain actions. They also enable a significant reduction in energy consumption. However, the training of SNNs is a challenging problem, strongly limiting their deployment. We then briefly overview new insights provided by the concept of a high-dimensional brain, which has been put forward to explain the potential power of single neurons in higher brain stations and deep SNN layers. Finally, we discuss the prospect of implementing neural networks in memristive systems. Such systems can densely pack on a chip 2D or 3D arrays of plastic synaptic contacts directly processing analog information. Thus, memristive devices are a good candidate for implementing in-memory and in-sensor computing. Then, memristive SNNs can diverge from the development of ANNs and build their niche, cognitive, or reflective computations.

## Introduction

### Brief History of Artificial Neural Networks

Since the early steps of artificial intelligence (AI), there have been several moments in history when it approached neuroscience in searching for bio-inspiration. In the middle of the twentieth century, the biomimetic approach was critical for developing artificial neural networks (ANNs). In 1943, W. McCulloch and W. Pitts proposed a model of the first artificial neuron (McCulloch and Pitts, 1943). The neuron received several synaptic-like inputs and generated an output if the number of activated synapses exceeded a threshold, thus mimicking the “all-or-none” principle of action potentials. Later, F. Rosenblatt further developed this idea and coined the term perceptron (Rosenblatt, 1958). Subsequent studies have shown the unreasonable effectiveness of artificial neurons coupled into networks in a constantly growing number of AI applications.

Mathematically speaking, an ANN is a function *y* = *f*(*x*) that maps input data, *x*, into output, *y*. Thus, it can emulate reflex responses. For example, to decide if there is a cat or a dog on an image, we can designate the image as the input *x*, and the animal category as *y*. Then, by presenting a photo to the ANN, we could quickly determine which animal appears in the image. But are we sure that there is such an ANN? In other words, can ANNs approximate arbitrarily complex functions?

The universal approximation theorem provides the answer. In 1989, G. Cybenko showed that an ANN with sigmoid activation could approximate any continuous function (Cybenko, 1989). Later, this result was extended into Lebesgue integrable functions and the rectified linear unit (ReLU) activation function (Lu et al., 2017; Hanin, 2019). In practical terms, no matter what *f*(*x*) is, there is an ANN approximating it with an arbitrary degree of accuracy.

However, mere existence is not enough for AI applications. The input and output sets and the function *f*(*x*) can be arbitrarily complex and high-dimensional. It makes impracticable, the use of human-tailored standard techniques like extracting a set of predictive features by principal component analysis, Fourier transform, etc. The main advantage of ANNs is the ability to learn from data, although such learning is data-hungry.

The synaptic weights of each neuron (i.e., the parameters of function *f*(*x*)) can be adjusted by a training process aiming at minimizing the prediction error. The optimization is usually done by a version of the stochastic gradient descent method (Robbins and Monro, 1951) based on the derivatives of the loss function evaluated by the backpropagation algorithm (Rumelhart et al., 1986). Thus, an ANN can automatically identify the hidden features essential for the classification. Then, the trained ANN can predict the output for unseen inputs taken from the same distribution, i.e., it gains the generalizing capability.

While feedforward fully connected ANNs were achieving solid results in many areas, until recently, they have been ineffective in tasks that are comparatively easy for humans (Schmidhuber, 2015). In 1997, Deep Blue, a chess machine developed by IBM, defeated the world champion, Garry Kasparov, while those days computers could not compete with kids in recognizing faces. This problem was utterly complex for AI systems, much harder than chess. To process an RGB photo with a rather mediocre resolution of 1 Mpx, the input layer of an ANN must have 3 × 10^6^ neurons. If the second layer has only 1,000 neurons, we approach 10^10^ synaptic weights to train. Thus, we rapidly get numbers prohibitive for modern computers, databases, and algorithms.

A critical breakthrough has been achieved by copying the converging architecture of the visual system of the brain (Olshausen and Field, 2004). In a seminal work, LeCun et al. (1989) reported a new class of ANNs, convolutional neural networks (CNNs; Goodfellow et al., 2016). The CNN architecture mimics the primate’s visual cortex (Hubel and Wiesel, 1968; Laskar et al., 2018). The V1 and V2 cortex regions are similar to convolutional and subsampling layers of a CNN, whereas the inferior temporal area resembles the higher layers (Grill-Spector et al., 2018; Khan et al., 2020).

Different filters using convolution have been known for a long time in image processing. But CNNs offered the possibility of learning these filters from the data automatically. As in the visual cortex, the first CNN layers detect simple shapes, such as lines and circles, and combine them into more complex features at each successive layer. The detected features stop making sense for a human observer at some point, but they encapsulate the essence of images (Altenberger and Lenz, 2018).

Thus, the rise of CNNs provided a methodology that allowed for outperforming humans in object recognition. In 2012, the AlexNet was the first CNN that beat traditional geometric approaches in the object recognition contest (Krizhevsky et al., 2012; Russakovsky et al., 2015). Since then, CNNs have constantly improved the results of the state-of-the-art (Altenberger and Lenz, 2018). The current winner, CoAtNet-7 (Dai et al., 2021), provides 90.88% of the Top 1 accuracy in image classification on the ImageNet benchmark (Imagenet, 2022). Although CNNs achieved superhuman performance in the visual pattern recognition in controlled competitions, humans are still much better in general recognition tasks (Schmidhuber, 2015).

### From Reflex to Reflective Neural Networks Aiming at Cognition

Artificial neural networks are already widely used in the first domestic robots, cars with an increasing autonomy to make in-driving decisions, and apps that manage our data and anticipate our actions and desires in daily life (Mackenzie, 2013; Lee et al., 2016; Bogue, 2017; Hussain and Zeadally, 2018). However, along with these successes, there emerges an awareness of fundamental limitations, mainly associated with the reflex nature of ANNs and shortcuts in simulating deep cognition, i.e., the mental process ruling our interactions with the environment. In the late 80s, Moravec (1988), Minsky, and others anticipated the unexpected slow progress in deep artificial cognition. The Moravec paradox says: *It will be much easier to create a robot capable of talking with us than a robot ready to move among us.* After almost 40 years, we can only confirm the prophecy.

Experimental studies on rats have shown that reinforcement is not necessary for learning (Tolman and Honzik, 1930). Rats actively process information rather than operate on a stimulus-response relationship as most contemporary ANNs do. Based on these data, in 1948, Edward Tolman coined the term cognitive map, which is an internal representation of one’s environment. Such an internal representation emerges as a reflective (thinking) processing of external information. Recent advances provided evidence of time compaction performed by the human brain when dealing with dynamic situations (Villacorta-Atienza et al., 2021). Theoretically, such compaction occurs through an active wave propagating in a neuronal lattice (Villacorta-Atienza et al., 2010, 2015). Thus, the fundamental difference between the biological neural networks in higher brain stations and modern ANNs is reflective vs. reflex information processing.

Future ANNs will also address the issue of energy efficiency. In modern ANNs, the information flow occurs continuously, and usually, all neurons are active and consume energy. Implementations of ANNs on GPUs are “hot ovens,” much hungrier for energy than the biological brains. Current trends in chip building go by increasing the power density (now about 100 W/cm^2^ vs. 0.01 W/cm^2^ for the brain) and the clock frequency (about 10 GHz vs. 10 Hz; Merolla et al., 2014). In the brain, only a tiny fraction of neurons are active at a given time. Neurons efficiently communicate by brief spikes and often remain quiet. Therefore, spiking neural networks (SNNs) mimicking real neurons progressively gain importance. However, the intrinsic complexity of SNNs slows down their expansion. In practical applications, current SNNs trained by supervised learning algorithms have already caught up with ANNs in recognition tasks (Shrestha and Orchard, 2018; Zambrano et al., 2019; Panda et al., 2020; Yin et al., 2021; Zenke and Vogels, 2021; Chen et al., 2022). However, the use of SNNs within the reflex paradigm limits the range of tasks to be solved and our understanding of the brain. We foresee that the future of SNNs will concentrate on the development of cognitive devices based on novel mathematical paradigms beyond standard ANN applications.

The recent experimental discovery of concept cells (Quian Quiroga, 2012) and the associated mathematical concept of a high-dimensional brain (Gorban et al., 2019) can boost the use of SNNs in tasks related to reflective information processing. Reflective SNNs can take advantage of their intrinsic dynamics and emulate complex, not reflex-based, brain actions, such as generating new abilities from previously learned skills. SNNs can be implemented as analog computational systems. We foresee that such systems will use the emerging memristive hardware paradigm for this purpose.

Memristors are passive elements of electrical circuits that can be densely packed in 2D or 3D matrices on a chip and emulate plastic changes in synaptic contacts in ANNs (Strukov and Williams, 2009; Jo et al., 2010). This enables a natural implementation of the synaptic integration of information in neurons. Thus, memristive crossbars are good candidates for building in-memory calculations for future reflective neural networks. The latter may open new horizons for deploying compact, low-power wearable devices that will provide a next-level cognitive experience to a user.

## Spiking Neural Networks as an Alternative for Building Reflective Artificial Intelligence

### Models of Spiking Neurons

The synergy between neuroscience and novel mathematical approaches can be a solution for building novel systems exhibiting reflective AI. In contrast to formal neurons used in ANNs, biological cells exchange information by brief pulses, called action potentials or spikes. Then, complex internal dynamics of neurons can significantly affect the processing and transmission of information, and the spike times matter.

Many mathematical models of spiking neurons have been proposed (Hodgkin and Huxley, 1952; FitzHugh, 1961; Koch and Segev, 1999; Izhikevich, 2003). They differ in the degree of biological realism. The most complete models use the Hodgkin–Huxley (HH) formalism. However, such models are computationally demanding, and their analytical analysis is complicated. In many practical applications, one can use an HH model only with the leaky current and assume that a neuron fires a spike if its membrane potential crosses a threshold. This reduction yields the simplest leaky integrate-and-fire model of spiking neurons (Abbott, 1999). Its most significant disadvantage is a reduced repertoire of dynamic behaviors, e.g., the absence of neuronal adaptation. However, if biological relevance is of no concern, integrate-and-fire models are attractive for large-scale simulations (Delorme et al., 1999). The Izhikevich model provides a balance between the computational cost and the variety of behaviors it can reproduce (Izhikevich, 2005). Besides modeling the neuronal membrane, there is a class of models, called multicompartmental, that also simulate the neuron’s morphology (Bower and Beeman, 1998; Koch and Segev, 1999). Such models are essential for studying complex processes occurring in a neuronal tissue, e.g., the spreading of depression or migraines (Makarova et al., 2010; Dreier et al., 2017).

### The Challenge of Training Spiking Neural Networks

Spiking neural networks are arguably more biologically realistic than ANNs, and the only viable option if one wants to simulate brain computations. Nevertheless, the reverse side of the coin is intrinsic complexity. The output of a neuron is no longer a univocal function of the input, which in turn is a fundamental property of a reflective system. Training SNNs usually employs diverse forms of supervised, unsupervised, or reinforcement learning. Different versions of Hebbian learning, particularly spike-timing dependent plasticity (STDP), have shown significant potential in a variety of cognitive tasks. Being experimentally supported, STDP strengthens a connection if the postsynaptic neuron generates a spike after the presynaptic one and weakens in the opposite case (Markram et al., 1997; Bi and Poo, 1998; Sjöström et al., 2001). We note that this type of plasticity has inherent elements of synaptic competition, which makes the “success” of the synapse dependent on the spike timings (Song et al., 2000).

Most modern attempts in training SNNs are still based on algorithmic approaches working well in ANNs, e.g., the minimization of loss (error) functions (Taherkhani et al., 2020). The so-called ANN-to-SNN conversion adopts methods already existing in deep ANNs. First, a corresponding ANN is trained, and then, taking into account some restrictions, the obtained synaptic weights are transferred to a similar SNN (Cao et al., 2015; Esser et al., 2016). Under this approach, the firing rates of spiking neurons should match the graded activations of formal neurons. Various optimization techniques and theoretical generalizations of this approach have been proposed (Diehl et al., 2015; Ruckauer et al., 2017).

In image processing, ANN-to-SNN methods allow for obtaining high accuracy, close to the performance of classical deep learning in ANNs (Neil et al., 2016; Tavanaei et al., 2019). When using event-based input data, e.g., from dynamic vision sensors, and energy-efficient hardware implementation, such SNN-based solutions can compete with deep ANNs (Cao et al., 2015; Esser et al., 2016).

Another approach to training SNNs relies on adapting the backpropagation algorithm to the temporal coding scheme in which input and output data are represented by relative spikes’ times (or delays). Several backpropagation-like algorithms for multilayer SNNs have been proposed, such as SpikeProp (Bohte et al., 2002), backpropagation with momentum (Xin and Embrechts, 2001), Levenberg–Marquardt algorithm for SNNs (Silva and Ruano, 2005), QuickProp and Resilient propagation (RProp) versions of SpikeProp (McKennoch et al., 2006; Ghosh-Dastidar and Adeli, 2007), and SpikeProp based on adaptive learning rate (Shrestha and Song, 2015). Mostafa (2018) used a transformation of variables in a feedforward SNN and showed that the input–output relation is differentiable and piecewise linear in a temporal coding scheme. Thus, methods of training ANNs can be used in SNNs. In the proposed back propagation-based algorithm, the performance of the SNN was slightly inferior to ANN. Still, it showed a much shorter time in the network response to a pattern presented to the input.

These approaches use only the first spike of each neuron during SNN learning and operating (the so-called time-to-first-spike or TTFS coding). Such limitation is overcome by methods using neurons capable of learning to fire a precise temporal spike pattern in response to a particular sequence of spike trains at the input: ReSuMe (Ponulak, 2005; Ponulak and Kasiński, 2010), tempotron (Gütig and Sompolinsky, 2006), chronotron (Florian, 2012), and SPAN (Mohemmed et al., 2012). These algorithms use biological-like elements in learning rules (such as STDP and anti-STDP window), but they work only with one (output) layer of spiking neurons or even with a single neuron. Further development of this idea offered supervised learning methods for multilayer networks with hidden neurons (Sporea and Grüning, 2013; Taherkhani et al., 2018).

Recently, the concept of surrogate gradients in SNNs has addressed the problem of the discontinuous derivative of the spike functions (Neftci et al., 2019). In particular, the spike function was approximated by a continuous one that served as the surrogate for the gradient. This approach enables direct training of deep SNNs using input spikes both in the temporal and rate coding schemes. The effectiveness of surrogate gradients in training deep SNNs achieved the state-of-the-art performance for an ANN in a significant number of standard tests (Shrestha and Orchard, 2018; Lee C. et al., 2020; Panda et al., 2020; Yin et al., 2021; Zenke and Vogels, 2021).

### Collective Dynamics in Spiking Neural Networks: Architecture vs. Function

Communication by spikes *via* plastic synaptic contacts provides different encoding modalities, including successive excitation, number of spikes in a train, spike timings (or phases) relative to a clock signal, rate encoding, etc. Various learning schemes for SNNs employ a binary categorization of processed information (Taherkhani et al., 2020; Dora and Kasabov, 2021). In other words, SNNs are initially thought of as biological neuron-like analog processing units that operate with digital computing tasks and tools.

Analog units with theoretically unlimited degrees of freedom are hardly controllable. Therefore, existing SNNs frequently lose in competition with modern ANNs originally constructed as algorithmic digitized networks solving logic computational tasks. However, reflecting SNNs mimicking structural and functional features of brain circuits have untapped the potential in exploring cognitive tasks, thereby bringing un closer to “intelligent” AI. To explore this potential, one should try to employ concepts of modern neuroscience from cellular and molecular to cognitive levels. Let us now have a short excursion into the concepts of structural and functional plasticity, which might be helpful in training SNNs to process data in a biologically relevant way.

In the brain, neuronal plasticity plays a crucial role in establishing functions. In common words, plasticity is an activity-dependent change in the dynamics of neurons and synapses at cellular (local) and circuit (global) levels. Features of the local synaptic plasticity typically appear as a change of synaptic strength depending on the local activity of corresponding neurons. The Hebbian learning rule is represented by STDP which corrects the synaptic strengths depending on spiking times between the pre- and postsynaptic neurons (Morrison et al., 2008). These changes may facilitate or depress particular signal transmission pathways in an unsupervised manner. In other words, STDP results in the formation of specific synaptic network architecture reflecting current activity patterns and, hence, may be specific to input data.

**FIGURE 1:**
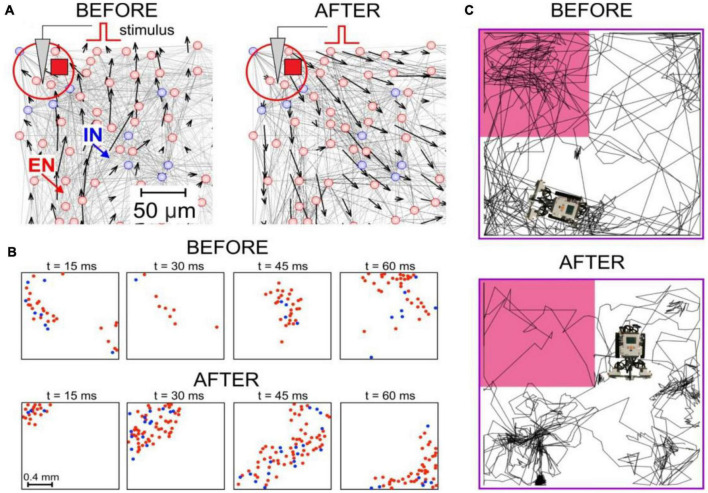
Spike-timing dependent plasticity (STDP)-driven spatial computing in spiking neural networks (SNNs). **(A)** The synaptic vector field of an SNN reveals the potentiation of centrifugal connections after local stimulation. **(B)** Connectome rearrangements lead to the transformation of patches of spike activity into traveling waves. **(C)** A neurorobot driven by an SNN avoids the dangerous zone (marked by pink) after learning.

At the circuit level, plastic changes can lead to different behaviors. The vector field method can be used to visualize the network architecture and functionality (Ponulak and Hopfield, 2013; Lobov et al., 2016, 2017). Figure 1A shows an example of the network reorganization provoked by a stimulus. Recently, it has been shown that there is an interplay between the anatomic architecture and functionality, and functional changes can drive the rebuilding of the network and vice versa (Lobov et al., 2021b).

Experimental data suggested that propagating patches of spike activity (Figure 1B) can play the role of basic functional units in brain information processes (Gong and Van Leeuwen, 2009; Muller et al., 2018). Based on this hypothesis, the concept of spatial computing was proposed, which can be defined as computations in neural networks mediated by the interaction of waves and patches of propagating excitation. This coding principle enables the detection of different signals and performing various stimulus transformations, for example, signal frequency reduction (Villacorta-Atienza and Makarov, 2013). One of the implementations of this concept can be considered a learning model in a neural network based on the STDP association of interacting traveling waves (Alexander et al., 2011; Palmer and Gong, 2014). Spatial computing in small neural circuits and modular SNNs can simulate Pavlovian conditioning and operant learning in neurorobots (Lobov et al., 2020b,2021a). Another possible way to implement spatial computations is cognitive maps and spatial memory with positive (Ponulak and Hopfield, 2013) or negative (Lobov et al., 2021b) environmental stimuli (Figure 1C). Note that due to the presence of spontaneous activity in SNNs (unlike ANNs), they can “live” without external input, determining the “behavior” of neurorobots (Lobov et al., 2020b,2021a,2021b).

The formation of cognitive maps and extraction of information from them can be based on the dependence of wave propagation on the connectom (Keane and Gong, 2015; Naoumenko and Gong, 2019; Lobov et al., 2021c). On the other hand, there are mechanisms for rapid switching of wave dynamics based on the balance of inhibition and excitation (Heitmann et al., 2012). Generalized cognitive maps provide another example of wave computations. In particular, the propagation of a wave of excitation in an SNN generates a cognitive map of a dynamic situation observed by a subject in the environment (Villacorta-Atienza et al., 2010; Makarov and Villacorta-Atienza, 2011).

Several studies have used unsupervised Hebbian learning in the STDP form to solve classification problems (Querlioz et al., 2013; Diehl and Cook, 2015; Tavanaei and Maida, 2015). Elements of reward in SNNs can force learning in a desirable direction (Izhikevich, 2007; Chou et al., 2015; Mozafari et al., 2018). Methods of supervised SNN learning are also proposed based on both temporal and frequency coding by stimulating target neurons (Legenstein et al., 2005; Lobov et al., 2020a). Another way to implement supervised learning is feedback from output neurons and element associative learning (Lebedev et al., 2020).

### Interplay of Neurons and Glial Cells in Spiking Neural Networks

In recent decades, experimental findings in cellular and molecular neuroscience revealed that glial cells also participate in information processing (Santello et al., 2019). Glial cells, specifically astrocytes accompanying neural networks, can effectively modulate local synaptic transmission (Perea and Araque, 2007; Durkee and Araque, 2019). Neurotransmitters diffusing from the synaptic cleft and bounding to specific receptors expressed in the plasma membrane activate astrocytes. In turn, the latter release neuroactive chemicals, called gliotransmitters, that activate specific receptors on both pre- and postsynaptic neurons. Such an interplay changes the efficacy of synaptic transmission on neighboring synapses. The modulation may last for dozens of seconds and have bidirectional influence, either facilitating or depressing synapses.

**FIGURE 2:**
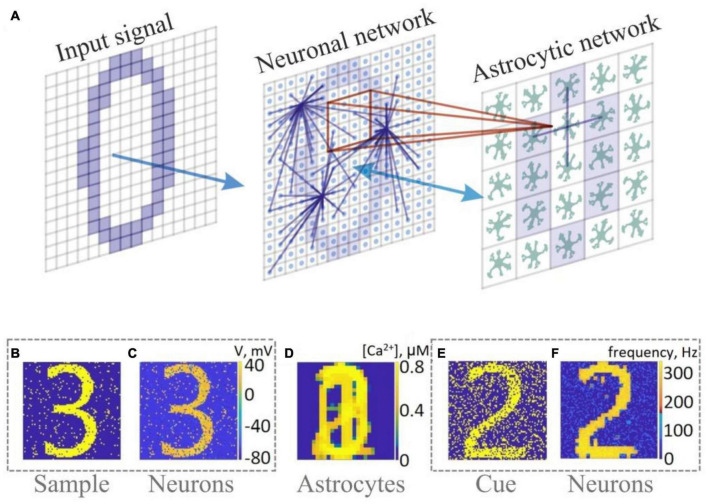
Memory enhancement in a neuron-astrocyte network. **(A)** Network topology. The SNN (79 × 79) consists of randomly coupled excitatory neurons. The astrocyte network (26 × 26) consists of diffusely connected cells. Blue lines show connections between elements in each layer. **(B–F)** Snapshots of training **(B–D)** and testing **(E,F)**.

Interacting with both pre- and postsynaptic neurons, astrocytes form a so-called tripartite synapse (Araque et al., 1999). In terms of information processing, astrocytes may enhance the learning capability of the network. Gordleeva et al. (2021) showed the possibility of memory enhancement by exploring an SNN interacting with astrocytes that served as reservoir preserving information patterns independently on neurons within dozens of seconds (Figure 2).

Local changes of synaptic strength, due to, e.g., short-term plasticity, regulate signal transmission in a synapse depending on its activity, often referred to as homosynaptic regulations. There is also another type of regulation called heterosynaptic plasticity when other inactive synapses change their efficiency (Chater and Goda, 2021). Heterosynaptic plasticity has different forms working at a similar time scale as the Hebbian plasticity. It can lead to both long-term potentiation and depression (LTP and LTD) of synapses. Thus, it can also play a crucial role in learning-related changes. Understanding of molecular and cellular mechanisms of heterosynaptic plasticity remains fragmentary. At the functional level, astrocytes may provide coordination between different signal transmission pathways (Gordleeva et al., 2019). Being activated by one of the synapses, astrocytes may release gliotransmitters back to the active synapses and inactive ones located at different spatial sites.

### Homeostatic Plasticity Is Relevant for Learning in Spiking Neural Networks

In living neural networks, homeostatic plasticity sustains the physiological conditions of functioning and balance (Keck et al., 2017). It prevents neurons from hyper- and hypo excitations. Thus, it acts mainly opposite the Hebbian learning rule that potentiates synapses in an activity-dependent manner.

Learning of complex patterns by an SNN requires neuronal competition (i.e., competition of the “outputs” of the network) similar to the “winner-takes-all” rule: the winning neuron should selectively recognize the pattern that caused its activation (refer to Section “Novel Mathematical Principles for Spiking Neural Networks: Concept Cells and High-Dimensional Brain”). It can be achieved by lateral inhibition (Quiroga and Panzeri, 2013; Lobov et al., 2020a,b). In addition to the neuronal competition, it is necessary to implement synaptic competition (i.e., competition of the “inputs” to the network). It can be achieved directly (Bhat et al., 2011; Lobov et al., 2020b) or indirectly *via* the homeostatic plasticity and synaptic scaling (Keck et al., 2017; Turrigiano, 2017) or synaptic forgetting (Panda et al., 2018; Lobov et al., 2020a).

Mechanisms of homeostatic plasticity were thoroughly studied, including synaptic scaling, changing postsynaptic density, control of excitation/inhibition balance, sliding thresholds for LTP, and LTD induction in Hebbian plasticity (Keck et al., 2017). An interesting point in the homeostatic changes concerns the activity of the brain extracellular matrix (ECM; Dityatev et al., 2010). The ECM is an activity-dependent environment for SNNs affecting synaptic transmission by synaptic scaling on the postsynaptic side and ECM receptors on the presynaptic one. At the functional level, the ECM works at much longer time scales (hours or days) and may serve as a long-term reservoir containing memory traces (Kazantsev et al., 2012; Lazarevich et al., 2020).

At the network level, changes in network architecture are driven by structural plasticity (Yin and Yuan, 2015). It accounts for structural changes in the number of synaptic receptors expressed in the dendritic spines, the number of synapses, and the number of neurons. The structural plasticity implements two essential strategic functions: (1) Sustain homeostasis. For example, the number of inhibitory synapses can increase to compensate for hyperexcitation (Keck et al., 2017). (2) Enhance learning capabilities (Hellrigel et al., 2019; Calvo Tapia et al., 2020a; Rentzeperis et al., 2022). For instance, synaptic receptors and synapses can be additionally generated to extend a specific signal-transmitting channel. In other words, the network architecture becomes dynamic. A network can change its dimension depending on the activity and entrust tasks. Thus, structural plasticity is crucial for learning, and network robustness compensates for injuries and ill-functioning states.

## Novel Mathematical Principles for Spiking Neural Networks: Concept Cells and High-Dimensional Brain

### How Does the Brain Encode Complex Cognitive Functions?

As we mentioned in the Introduction, the brain is not inert but actively generates predictions about what will happen next. Such predictions presumably occur in higher brain stations that summarize and process converging information from different sensory pathways. An intriguing question concerns the role of individual neurons in complex cognitive functions and, in the end, in conciseness. This question is as old as Neuroscience itself. It yielded many significant results, such as discovering efficient or sparse coding (Barlow, 1961; Field, 1987; Olshausen and Field, 1997), and is far from being satisfactorily answered (Valdez et al., 2015).

A somewhat extended opinion says that complex intellectual phenomena result from a perfectly orchestrated collaboration between many cells (Bowers, 2009). This idea, known as the “million-fold democracy,” was put forward by C. Sherrington (1940). Our actions are driven by the joint activity of millions of neurons in an “election” in which some neurons vote more often than others. It yielded the concept of population coding: The brain encodes information by populations or clusters of cells rather than by single neurons (Pouget et al., 2000). For example, in the primate primary motor cortex, individual neurons are tuned to the direction of arm movement, and populations of such neurons have to be pooled together to compute the direction a monkey is about to move its arm (Georgopoulos et al., 1986). This finding prompted the development of brain–machine interfaces using population coding (Lebedev and Nicolelis, 2017).

In 1890, even before the pioneering works on neuroanatomy by S. Ramon y Cajal, W. James (1890) proposed that neurons have individual consciousness and that there is one “pontifical” cell to which our consciousness is attached. Although such an idea sounds absurd nowadays, it may not be so far from the truth. According to J. Edwards (2005), to combine different flows of information into a smoothly unrolling, multi-modal experience of reality, the relevant bits of information must come together in one unit somewhere. A brain or its parts are too big, but a single neuron may be just about right. Gnostic (i.e., single-cell) coding may also provide metabolic efficiency. The high cost of spiking drives the brain to use codes that minimize the number of active neurons (Lennie, 2003).

### Individual Concept Cells Can Be Responsible for Cognitive Phenomena

The “degree” of consciousness in gnostic cells may depend on the spatial pattern a neuron receives (Sevush, 2006; Cook, 2008). The conscious activity of neurons in the initial relay stations is simple and cannot directly affect the animal’s macroscopic behavior. However, at higher brain stations, neurons operate with complexity and diversity sufficient to account for complex conscious experiences. Converging experimental evidence confirms that small neuron groups or single cells can implement complex cognitive functions, such as generating abstract concepts.

Some pyramidal neurons in the medial temporal lobe (MTL) can exhibit remarkable selectivity and invariance to complex stimuli (Quian Quiroga et al., 2005; Mormann et al., 2011). It has been shown that the so-called concept cells (or grandmother cells) can fire when a subject sees one of seven different pictures of Jennifer Aniston but not the other 80 pictures of other persons and places. Concept cells can also fire to the spoken or written name of the same person (Quian Quiroga, 2012). Thus, a single concept cell responds to an abstract concept but not to the sensory features of the stimuli. Moreover, concept cells are relatively easily recorded in the hippocampus (Quian Quiroga, 2019). Thus, they must be abundant, at least in the MTL, contrary to the common opinion that their existence is highly unlikely (Bowers, 2009). Kutter et al. (2018) have found that single neurons in MTL encode numbers. They suggested that number neurons provide the neuronal basis of human number representations that ultimately give rise to number theory and mathematics.

### Spiking Neural Networks Can Take Advantage of the Blessing of Dimensionality

**FIGURE 3:**
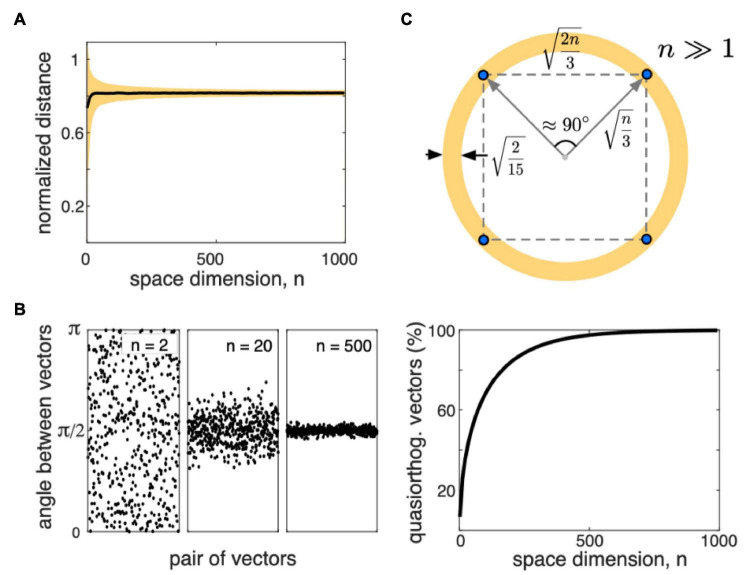
Some properties of the distributions of random vectors in high-dimensional (HD) spaces (*n* is the space dimension). **(A)** The mean (black) and standard deviation (yellow) of the distance between two randomly chosen points sampled from a uniform distribution in the hypercube [−1, 1]^*n*^, normalized to $\sqrt{n}$. Distances concentrate around $\sqrt{2\,n/3}$. **(B)** *Left:* Examples of the angles between pairs of randomly chosen vectors for *n*2, 20, and 500. For high *n*, angles concentrate around π/2. *Right:* Proportion of quasi-orthogonal (with the tolerance 0.1 rad) vectors vs. the space dimension. For high enough dimensions, almost all vectors are quasi-orthogonal. **(C)** Sketch of the distribution of random points (blue dots) in a high-dimensional space.

The discovery of concept cells has stimulated theoretical research, which led to the theory of a high-dimensional brain (Tyukin et al., 2019). It uses fundamental properties of high-dimensional (HD) data. On the one hand, in HD-spaces, we can observe the curse of dimensionality, the term coined by Bellman (1957). It highlights, for instance, the combinatorial explosion. To sample *n* Boolean features, we must check 2^*n*^ cases. Even for a relatively low dimensional space with *n* = 30, this number goes to almost 10^10^, prohibitive for modern computers. Another example is the concentration of the distances between randomly selected points. If *n* increases, the pairwise distances concentrate around the mean value (Figure 3A). Then, the distance-based methods, such as k-nearest neighbors work poorly (Beyer et al., 1999; Pestov, 2013; Lin et al., 2014).

On the other hand, it turns out that rather general stochastic processes can generate HD signals with relatively simple geometric properties (Gorban et al., 2019, 2020). In 2000, D. Donoho introduced the term “blessing of dimensionality,” with which the curse of dimensionality are two sides of the same coin (Donoho, 2000). As a system becomes more complex, it has been observed that its analysis can be complicated at first, but then it becomes simpler (Kreinovich and Kosheleva, 2021). A good example is the Central Limit Theorem. A statistical analysis of a few random variables can be highly complicated. However, a mixture of many random variables follows a Gaussian distribution and can be easily described by the mean and the standard deviation.

Both the curse and the blessing of dimensionality are the consequences of the measure concentration phenomena (Ledoux, 2005; Gorban et al., 2016; Gorban and Tyukin, 2018). Figure 3B illustrates examples of the angle between two randomly chosen vectors (sampled from a uniform distribution in a hypercube [−1, 1]^*n*^). Together with the distribution of the inter-point distances, we can conclude that all vectors having approximately equal length, are nearly orthogonal, and the distances between data points are roughly equal (Figure 3B). Figure 3C illustrates a sketch of how random data points appear in the HD space. Tyukin et al. (2019) hypothesized that neurons could take advantage of such a notorious simplicity of the distribution and use simple mathematical mechanisms for processing complex, HD data.

### High-Dimensional Neurons Can Exhibit Unexpected Properties

According to Sevuch and Cook (see, e.g., Sattin et al., 2021), the synaptic connections within a neural network could represent the substrate of cognition. The pattern complexity plays a key role, and conscious human behavior requires the processing of complex multidimensional data. Recent empirical evidence shows that a variation in the dendrite length and hence in the number of synapses *n* can explain up to 25% of the variance in IQ scores between individuals (Goriounova et al., 2018).

**FIGURE 4:**
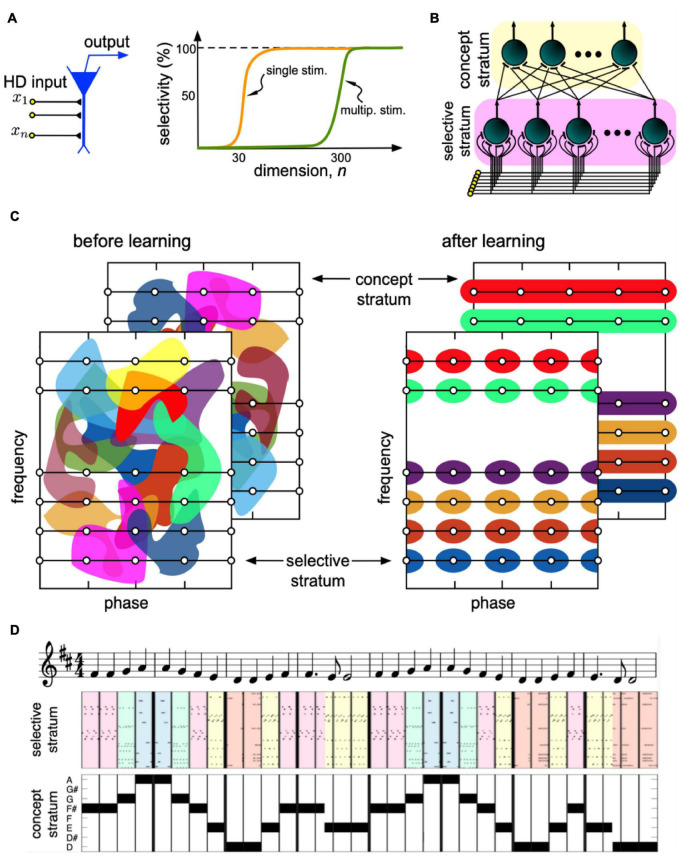
The concept of an HD brain. **(A)** A neuron receives an HD pattern *x* and produces output if the pattern matches the pattern of synaptic weights. The cognitive functionality of a neuron develops in steps. At dimensions *n* 30, the neuron becomes capable of selectively detecting a single stimulus from a large set (orange curve). At *n* 300, a new ability appears. The neuron can now detect multiple uncorrelated stimuli. **(B)** Model mimicking the information flow in the hippocampus. A stimulus, e.g., a sound wave, goes to HD neurons in the selective stratum. Neurons learn different sound waves and send collaterals to neurons in the concept stratum. Concept cells extract concepts of musical notes. **(C)** Under learning, the rearrangement of the neuronal receptive fields leads to the formation of note-specific concept cells (different colors correspond to the receptive fields of different neurons). **(D)** Binding of sounds into notes for a fragment of the 9th Symphony by Beethoven “Ode to joy”.

Figure 4A illustrates a theoretical model of an HD neuron (Tyukin et al., 2019). The neuron receives as an input an HD vector pattern ***x*** = (*x*_1_, …, *x*_*n*_)*^T^* ∈ [−1, 1]*^n^*, such that the number of individual inputs or the neuronal dimension *n*≫1. The inputs are connected to the neuronal membrane through synaptic contacts. The output of the neuron is given by a transfer function, e.g., ReLU. During operation, the synaptic weights of the neuron change by a Hebbian-type rule (Calvo Tapia et al., 2020a).

Given that the distribution of the input patterns in a big but finite set of stimuli has no strong clots, it has been shown that the neuron can accurately learn a single pattern from the entire set. An important consequence is that no a *priori* assumptions on the structural organization of neuronal ensembles are essential for explaining the fundamental concepts of static and dynamic memories. Cognitive functionality develops with the dimension of single neurons in a series of steps (Gorban et al., 2019; Tyukin et al., 2019).

The neuronal selectivity emerges when the dimension exceeds some critical value, around *n* = 30 (Figure 4A). At this crucial transition, single neurons become selective to single information items. The second critical transition occurs at significantly larger dimensions, around *n* = 300. At this second stage, the neuronal selectivity to multiple uncorrelated stimuli develops. The ability to respond selectively to a given set of numerous uncorrelated information items is crucial for rapid learning “by temporal association” in such neuronal systems.

### Single High-Dimensional Neurons in Deep Spiking Neural Network Layers May Provide Cognition

Remarkably, a simple generic model offers a clear-cut mathematical explanation of a wealth of empirical evidence related to *in vivo* recordings of “grandmother” cells and rapid learning at the level of individual neurons. It also sheds light on the question of why Hebbian learning may give rise to neuronal selectivity in the prefrontal cortex (Lindsay et al., 2017) and explain why adding single neurons to deep layers of ANNs is an efficient tool to acquire novel information while preserving previously trained data representations (Draelos et al., 2017).

Calvo Tapia et al. (2020b) extended results into the problem of building abstract concepts by binding individual items of the same kind. Figure 4B illustrates the model mimicking primary signaling pathways in the hippocampus. It considers the stratified structure of the hippocampus that facilitates ramification of axons, leaving multiple buttons in the passage and conveying the same HD input to multiple pyramidal cells (Teyler and Discenna, 1984). The latter has been supported by electrophysiological observations showing that Schaffer collaterals create modules of coherent activity with a large spatial extension in the CA3 region (Benito et al., 2014, 2016). Thus, the hippocampal formation possesses rather exclusive anatomical and functional properties required for the emergence of concept cells.

In the beginning, the receptive fields of all neurons (areas in the sensory domain evoking a response) in both strata form a disordered mixture of random regions (see the cartoon in Figure 4C, left). Thus, the output of the concept stratum is random, and the system cannot follow the music. The purpose of learning is to organize the receptive fields so that the concept cells become note-specific (Figure 4C, right). In this case, each concept cell will not be stimulus-specific but represent a set of associated stimuli or a concept, e.g., note A.

The network has been tested on the perception of the 9th Symphony by Beethoven (Figure 4D). The selective stratum detects individual sound waves, while the concept stratum puts them together and forms the note-specific output (Calvo Tapia et al., 2020a). Thus, concept cells respond to particular notes regardless of the phase of sound waves, and the “brain” now does follow the music. This result supports the hypothesis of a strong correlation between the level of neuronal connectivity in living organisms, and different cognitive behaviors such organisms can exhibit (Herculano-Houzel, 2012).

## The Memristive Architecture Enables the Implementation of Reflective Spiking Neural Networks

### What Is a Memristor?

In 1971, Leon Chua (1971) discovered the memristor as a hypothetical fourth passive element of electrical circuits. A memristor relates a change in the magnetic flux with a variation of the electric charge flowing through this element. Mathematically, it is equivalent to a nonlinear resistor that changes its resistance depending on the history of the electric current. Therefore, it was called a memristor, i.e., a memory resistor.

In 2008, Strukov et al. (2008) associated the memristive effect with resistive switching in thin-film metal-oxide-metal structures. Such films were actively studied as early as the middle of the twentieth century (Dearnaley, 1970). Starting in 2008, the current wave of interest in memristors began to rise. Although memristors have been thoroughly studied, there are still debates and doubts about the existence of an ideal memristor satisfying the original definition and the validity of its correlation with resistive switching (Vongehr and Meng, 2015; Demin and Erokhin, 2016; Kim et al., 2020). Despite that, the generalized definition of a memristor as a dynamical system, which Chua and Kang (1976) proposed in 1976, remains valid. According to it, a memristor is a system described by the following equations:


(1a) $$I\,\left(t\right)=\frac{V\,\left(t\right)}{R\,\left(x,V\right)}$$

(1b) $$\frac{d\,x}{d\,t}=f\,\left(x,V\right),$$

where *I*(*t*) is the current flowing through the system, *V*(*t*) is the voltage drop, *R*(*x*,*V*) is the resistance with memory or memristance, *x*(*t*) ∈ ℝ^*m*^ is an *m*-dimensional dynamic variable describing the internal state of the system, and *f* : ℝ^*m*^ × ℝ → ℝ^*m*^ is a nonlinear function.

From a physical point of view, Equation 1 is Ohm’s law, which describes any nonlinear memory resistor, regardless of the nature of the nonlinearity and the mechanism of resistance change. Thus, the generalized definition (1) of the memristive effect applies to the description of resistive switching in any materials: inorganic (Ohno et al., 2011), organic (Demin et al., 2014), molecular (Goswami et al., 2020), etc. Various physical and chemical phenomena, including ion migration and redox reactions, ferroelectric and magnetoresistive effects, and phase transitions, can be responsible for the change in the resistance of inorganic materials and structures. Wang et al. (2020) provided a detailed comparison of different resistive switching mechanisms and concluded that resistive random-access memory (RRAM) are superior in terms of dimension (nanometer-scale), number of distinguishable resistive states (>64), switching speed (picoseconds), endurance (10^12^ cycles), and retention (10^3^ years).

**FIGURE 5:**
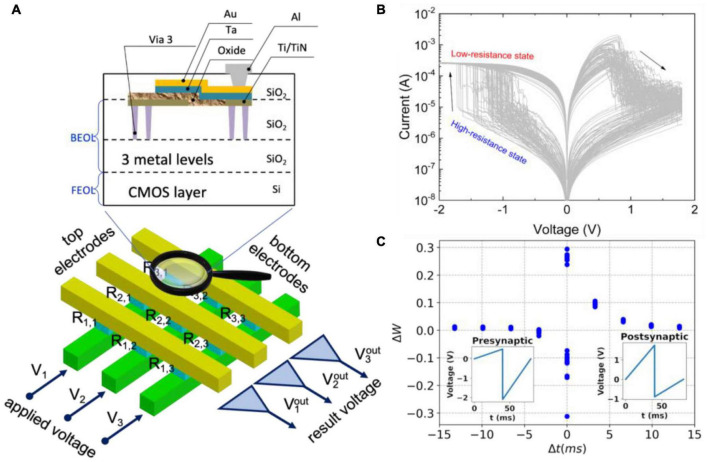
Memristive systems. **(A)** An array of crossbar metal-oxide-metal memristive devices integrated into the top metallization layers back-end-of-line (BEOL) of the CMOS layer front-end-of-line (FEOL). **(B)** Typical current-voltage characteristics of a memristive device with stochastic switching between low- and high-resistance states. **(C)** Synaptic functionality of the memristive device mimicking the STDP rule.

The metal-oxide-metal structures of the RRAM type (Figure 5A) are the most compatible materials to be integrated into the conventional CMOS process (Ielmini and Waser, 2016). Such devices can store Boolean values given by the conductivity and allow it to be changed in the same physical place, implementing new “non-von Neumann” paradigms of in-memory computation (Papandroulidakis et al., 2017; Erokhin, 2020; Lee S. H. et al., 2020). It is provided by the typical current-voltage characteristics with a pinched hysteresis (Figure 5B). It exhibits a wide range of resistances, as well as the pronounced and inherent stochastic nature of the conductance switching in memristors. The change in conductivity of a memristive device in response to spiking activity is analogous to the plasticity of a biological synapse and is usually described by the STDP rule (Figure 5C; Zamarreño-Ramos et al., 2011; Emelyanov et al., 2019; Demin et al., 2021).

The simple two-terminal structure of the memristor enables the building of superdense and, in future, three-dimensional “crossbar” arrays (Figure 5A). Based on Ohm’s and Kirchhoff’s laws, such arrays naturally implement analog operations of matrix-vector and matrix-matrix products, underlying the massive computations in traditional ANNs (Xia and Yang, 2019; Mehonic et al., 2020). Recently, using an analog-digital platform, it has been shown that a memristive crossbar can perform analog operations while digital circuits control the crossbar and enable writing synaptic weights into them (Bayat et al., 2018; Cai et al., 2019; Wang et al., 2019; Yao et al., 2020; Zahari et al., 2020). Thus, hardware-based ANN algorithms for learning and operating have been implemented based on memristors. They can significantly improve the parameters of neuromorphic computing systems, which have been actively developed in recent years due to new applications, algorithms, and element base (Indiveri et al., 2011; Schuman et al., 2022).

### Memristor as a Key Element in Building Reflective Spiking Neural Networks

**FIGURE 6:**
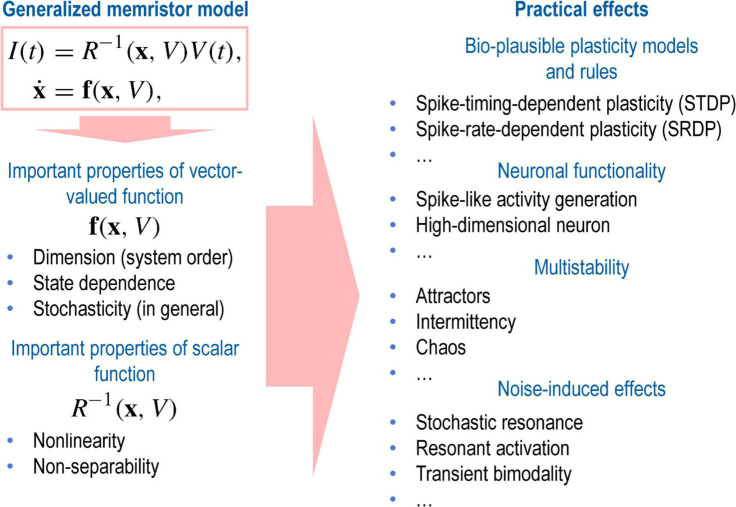
Schematic illustration of the variety of practical effects hidden in the basic properties of the generalized memristor model.

Let us now discuss the rich dynamics of memristive systems and present some examples within the framework of the above-mentioned conceptual approaches. The universal description of the memristor system expressed in Equation 1, hides a plethora of sound effects that yield various functional applications of memristors (Figure 6). The function *f*(*x*,*V*) plays a central role in the dynamics of a memristive system and determines the complexity of the internal state of the system (Pershin and Slipko, 2019a). Moreover, the function *f*(*x*,*V*) can include both internal and external noise, making it possible to describe a memristor as a stochastic system (Agudov et al., 2020).

Contrary to a popular belief and the standard approach focused on studying the state equation with a linear function, *f*(*x*,*V*) plays a decisive role in achieving the complex dynamics of a memristive system. Moreover, for complex dynamics, the memristance *R*(*x*,*V*) should be a nonlinear and a nonseparable function of its variables (Guseinov et al., 2021a). It yields the condition *R*(*x*,*V*)≠*g*(*x*)*p*(*V*). Different combinations of these desired properties enable simple or arbitrarily complex behaviors of real memristive devices.

Among the simple examples that can be obtained by using the first-order linear models, we can mention the widespread Hebbian plasticity described by the STDP rule (Figure 5C). It can be achieved by overlapping signals from pre- and postsynaptic neurons applied to a memristor (Zamarreño-Ramos et al., 2011; Emelyanov et al., 2019; Demin et al., 2021). More complex versions of plasticity, for example, frequency-dependent, require at least two dynamic variables operating at different time scales (Du et al., 2015; Kim et al., 2015; Matsukatova et al., 2020). Three state variables yield a neuron-like activity of a memristive device based on a volatile type of resistive switching (Kumar et al., 2020).

It is worth noting that the rich dynamics of memristive devices allows for going beyond the conditional rules of plasticity. We can build neural networks from the first principles based on the self-organization of adaptive memristive connections and the synchronization of neurons coupled by memristive devices. The coupling of neurons by the stochastic plasticity in memristive connections has been illustrated experimentally for several neurons in an ensemble (Ignatov et al., 2016, Ignatov et al., 2017; Gerasimova et al., 2017). The experimentally observed complex dynamics of memristively connected neurons requires description using high-order dynamical models to design larger brain-like cognitive systems (Gerasimova et al., 2021).

The use of a nonlinear potential function describing the state of memristors leads to the appearance of different types of attractors in the state space, which drives the dynamic characteristics of the memristors (Pershin and Slipko, 2019a,b). The multidimensionality of this space, combined with nonlinear and nonseparable memristance, provides the necessary and sufficient conditions for observing the complex dynamics of the memristor response to external periodic stimulation (Guseinov et al., 2021a). The corresponding transition from periodic response modes to intermittency and chaos can partially explain the variability in the parameters of real memristive devices (Guseinov et al., 2021b).

Stochasticity is an intrinsic property of a memristor (Carboni and Ielmini, 2019). Noise can be used both to study the multistable nature and to control the behavior, thanks to such well-known effects as stochastic resonance and enhancement of the stability of metastable states (Mikhaylov et al., 2021), resonant activation (Ryabova et al., 2021), etc. These and other phenomena related to the constructive role of noise can be described within the framework of analytical stochastic models (Agudov et al., 2020, 2021). They are well suited for design at the circuit level.

Thus, the presented range of functional capabilities of memristive systems already makes it possible to implement SNN architectures in hardware. Although memristor-based SNNs have already been developed and even tested in crossbars (Ankit et al., 2017; Prezioso et al., 2018; Demin et al., 2021), they roughly simulate STDP to implement local learning rules. However, STDP does not cover the whole variety of biochemical processes and describes only one of the mechanisms determining synaptic plasticity (Feldman, 2012). Moreover, the memristive STDP models use a simplified algorithm based on a temporal overlap of pre- and postsynaptic spikes at a millisecond time scale (Demin et al., 2021). They essentially can be reduced to the direct programming of the memristor resistive state. Such an approach significantly complicates the electric circuits of the developed SNNs and compromises their energy efficiency and performance. However, it is still relevant for building small-sized demonstration prototypes. We foresee further implementations of various mechanisms of synaptic plasticity, multistability, and stochasticity at the complexity level critical to building perfect systems from imperfect elements. At the same time, the immature memristive technology cannot currently meet the constantly growing requirements for ANNs from developing digital services. Large-scale crossbar arrays suffer from several parasitic effects.

In the section below, we overview two approaches that should reveal the potential of memristive devices in reflective (“thinking”) information and computing systems. They are being developed as alternatives to the standard “digital” approach based on programming the states of memristive devices as customarily done in traditional electronics. The first approach aims at creating self-learning SNNs based on the rich dynamics of memristive devices and simple architectures, using elegant and efficient solutions prompted by nature and corresponding to the well-known principle of simplicity in neurosciences (refer to Section “Novel Mathematical Principles for Spiking Neural Networks: Concept Cells and High-Dimensional Brain”). The second approach proceeds by completely rejecting digital algorithms and implies direct (on-site or at the edge) processing of analog information from outside. It aims to effectively implement such perception functions as vision, hearing, etc.

### Memristive High-Dimensional Neurons as Building Blocks for Artificial Cognitive Systems

The possibility of a mathematical description and hardware implementation of synaptic functions based on a memristive device enables implementation of even the most daring mathematical concepts in hardware. Recent advances use simplified architectures of neurons and include the concept of the high-dimensional brain (Section “Novel Mathematical Principles for Spiking Neural Networks: Concept Cells and High-Dimensional Brain”), which explains the unreasonable efficiency of single cognitively specialized neurons. The system consists of software and hardware parts and is controlled by a microcontroller (Shchanikov et al., 2021). Memristive devices based on a metal-oxide-metal thin-film structure, where yttrium-stabilized zirconium dioxide acts as a switching medium, can implement adaptable synaptic weights of a high-dimensional neuron (Mikhaylov et al., 2020).

**FIGURE 7:**
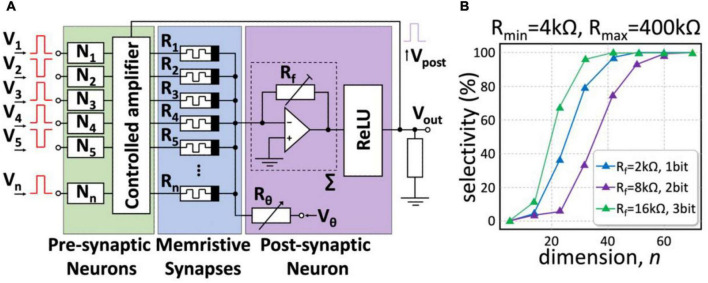
The concept of a memristive HD neuron. **(A)** Hardware implementation of an HD neuron. A neuronal membrane (purple) is an inverting adder receiving input from the synaptic connections made of memristors *R*_1_,…, *R*_*n*_ (blue). The feedback loop enhances couplings of pre- and post-neurons under learning through the controlled amplifier (green). **(B)** The result of simulations of the electric circuit. As the HD-theory predicts, the neuronal selectivity steeply increases with the dimension and attains 100% for *n* = 50 − 70.

Figure 7A illustrates an electric circuit implementing a high-dimensional (HD) neuron. An HD input vector pattern encoded by bipolar pulses *v* = (*V*_1_,…,*V*_*n*_)^*T*^ is fed to the circuit input. The resistances of the memristive devices *R*_1_,…,*R*_*n*_ determine the weights of the synaptic connections, and their combination for a particular neuron determines the neuron selectivity as discussed in Section “Novel Mathematical Principles for Spiking Neural Networks: Concept Cells and High-Dimensional Brain”. Then, an inverting adder implements the main functionality of the neuronal membrane by integrating *n* informational channels and the membrane threshold, *V*_θ_. Such a neuron performs mathematical operations of multiplication and addition following Ohm’s and Kirchhoff’s laws.

The proposed analog circuit implementation of an HD neuron is simple but, at the same time, allows for simulating the concept described in Section “Novel Mathematical Principles for Spiking Neural Networks: Concept Cells and High-Dimensional Brain”. Figure 7B shows the result of simulations of the operational performance of a memristive HD neuron with memristive devices working in the resistance range of *4-400* kOhm. At high dimensions, the neuron exhibits absolute selectivity to the input stimuli. Such neurons can be used to implement a variety of cognitive behaviors (Tyukin et al., 2019; Calvo Tapia et al., 2020b; see also Section “Novel Mathematical Principles for Spiking Neural Networks: Concept Cells and High-Dimensional Brain”). The switching dimension depends on the circuit components and starts at *n* = 50. By adjusting the value of *R_f_*, we can achieve absolute selectivity even for the relatively low resolution of neuron weights (Figure 7B).

Learning the proposed HD neuron is automatic and goes the following way (Calvo Tapia et al., 2020b). At the beginning, the memristors have arbitrary initial resistances, which means that the neuron has some combination of the synaptic weights. Then, we supply to the neuron a sequence of input data vectors encoded by the inverted voltage amplitudes of the input signal *v*. The maximal amplitude must not exceed the switching voltage of the memristors *V*_*th*_ to maintain the original combination of the resistances. At the presentation of a certain input vector, the ReLU output will become positive, which means the neuron has detected the vector. Then, following the Hebbian rule, the coupling of pre- and postsynaptic neurons is strengthened. It is achieved by setting voltages at the inputs of the neuron required to increase or decrease the resistance of the corresponding memristors in the range [*R*_*min*_,*R*_*max*_] for positive and negative inputs, respectively.

To train an HD neuron at the hardware level, we can use noninverting operational amplifiers with controlled gain (controlled amplifier in Figure 7A). The gain is adjusted by adding a load to the feedback of the operational amplifier when the voltage-controlled switch is opened. In the feedback loop, the voltage *V*_*post*_ is set at the ReLU output only when the neuron detects an input pattern, and this pulse opens the keys and increases the amplitude of the input pulses *V*_1_,…,*V*_*n*_. In turn, it causes a change in the resistance of *R*_1_,…,*R*_*n*_, and in the strength of the synaptic connections.

### Bio-Inspired Analog Signal Processing Enabled by Memristors

According to the second alternative approach, memristive devices and SNNs may also facilitate the implementation of neuromorphic analog machine vision systems. HP Labs and the University of Berkeley have shown one of the first implementations of an ANN with memristive devices used for pattern recognition. Bayat et al. (2018) described a device based on passive crossbars with 20 × 20 memristors, which implements a multilayer feed-forward perceptron capable of recognizing Latin alphabet letters with 97% accuracy.

A publicly available simulator of the human retina (Eshraghian et al., 2019; Baek et al., 2020) can be used to develop advanced analog vision systems. Based on computing systems with memristor chips, a Hopfield ANN and a convolutional ANN were implemented and tested in pattern recognition tasks and associative memory (Zhou et al., 2019; Li et al., 2020; Yao et al., 2020). It has been shown that the implementation of ANNs on memristive devices of the size 128 × 64 is several times faster than graphics and signal processors in terms of speed and lower power consumption (Li et al., 2018a,b). In general, the results of comparing memristive devices with modern systems of a hardware implementation of ANNs show their advantages in accuracy, speed, power consumption, etc. (Xia and Yang, 2019; Amirsoleimani et al., 2020; Lee S. H. et al., 2020; Qin et al., 2020).

At the same time, the need for analog-to-digital and digital-to-analog conversions minimizes the potential energy gain from using memristors in traditional architectures (Amirsoleimani et al., 2020). Memristive devices allow for creating neuromorphic systems in which all processing takes place in an analog form. Thus, it seems reasonable to exclude analog-to-digital and digital-to-analog conversions from machine vision systems. The signals from the photosensor can be fed to an SNN without digitization. Then, the conductivities of the memristors will shape the model of visual information processing and simultaneously perform this processing (in-sensor computing).

The first steps have already been taken to combine memristive devices with photosensors. The described architecture of a 1D1R sensor for machine vision is a 20 × 20 or 32 × 32 matrix of SiN_x_ memristive devices coupled to a photodiode or a phototransistor (Vasileiadis et al., 2021a,b). The coupling of memristors with photosensors shows that this approach can simulate some retinal functions (Chen et al., 2018; Eshraghian et al., 2018). Adding such photosensors to layers of SNNs based on memristors may allow for the implementation of the concept of analog machine vision.

**FIGURE 8:**
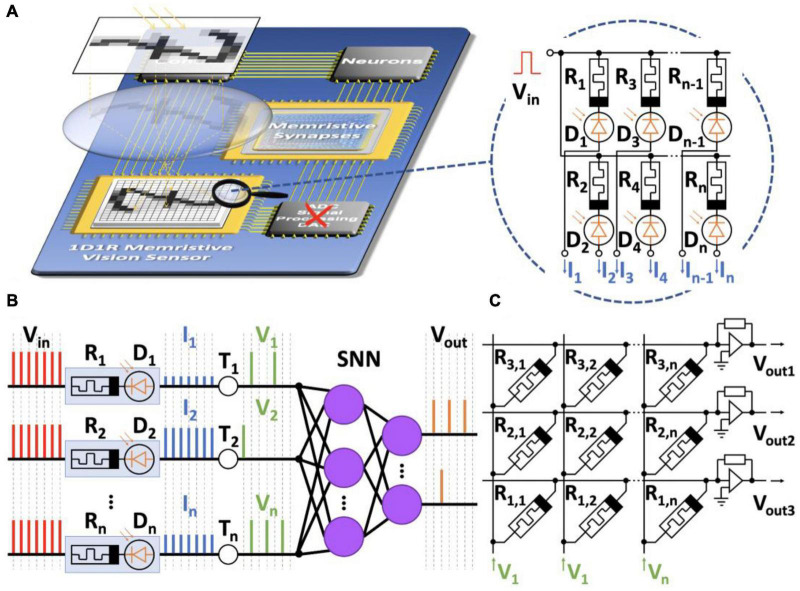
The concept of analog memristive vision. **(A)** Schematic representation of the vision system. It includes a 1D1R memristive sensor capturing images, synaptic connections (memristive crossbar), and an SNN. There is no need for an analog-to-digital converter (red cross). **(B)** Process of transformation of spikes in the memristive SNN. **(C)** A layer of memristive neurons implemented by an array of memristors in a crossbar.

Figure 8 illustrates the concept of analog memristive vision exploiting coupled memristors and photodiodes (Vasileiadis et al., 2021a,b). The 1D1R memristive sensor receives visual information (Figure 8A). The sensor is a photodetector consisting of photodiodes *D*_1_,…,*D*_*n*_ connected to a voltage source *V*_*in*_ and memristors of resistances *R*_1_,…,*R*_*n*_. The voltage source forms spikes at the input of the SNN. After exposure, the memristors change their resistances depending on the illumination. Therefore, a different voltage drop will occur in each input channel when voltage pulses are applied. Then, the first SNN layer consisting of integrate-and-fire neurons fires spikes with frequencies depending on the resistance of the memristors and the thresholds *T*_1_,…,*T*_*n*_ (Figure 8B).

Thus, visual information can be encoded by analog spikes without analog-to-digital conversions and transmitted directly to the input of the memristive SNN. The main element of the SNN is the memristive crossbar (Figure 8C). Memristors in the crossbar can change their conductivities and play the role of synapses. Since spikes come at different frequencies at the input, the STDP model can be used in the SNN to implement local learning rules.

We note that the concept of a high-dimensional brain and analog machine vision complement each other and may bring this area to a qualitatively new level. Although we have described only the simplest selective effect emerging in HD neurons, more complex architecture (see, e.g., Calvo Tapia et al., 2020b) are ready to be implemented in memristive architectures and SNNs.

## Conclusion

In recent decades, SNNs have increasingly gained attention. This study has provided an overview of current theoretical, computational, and hardware approaches to building reflective SNNs. Some of the discussed problems, such as learning in SNNs, are unsolved and require new efforts from the scientific community. The synergy between neuroscience and mathematical approaches can be a solution for building novel systems demonstrating reflective AI.

Current neural networks usually deal with the abstraction of “static” stimuli (objects, persons, landscapes, or even speech). The abstraction of actions and behaviors is a great challenge that should be addressed in the future. Some of the proposals argue that it can be done through a specific type of internal representation (Calvo Tapia et al., 2020c) or through building motor motifs (Calvo Tapia et al., 2018). Now our knowledge about higher echelons of information processing in the brain is limited. There is no clear evidence on how biological neurons represent spatiotemporal concepts and end up with cognition. However, it likely happens in an active manner through a constant interplay between the intrinsic brain dynamics and external input.

The theory of the HD brain, based on the measure concentration phenomena, suggests that individual neurons can become “intelligent” through a series of quantum leaps if the complexity of information they process grows. It helps explain that a cognitive phenomenon is not a linear combination of component functions. Adding up components increases the system dimension, and at some key points, novel faculties emerge. These advances suggest that learning in higher brain stations can be majorly local, and different versions of Hebbian rules, e.g., STDP, can be behind various cognitive phenomena.

The hardware friendliness of SNNs has stimulated the search for methods of their implementation in low-power hardware devices. We foresee that memristive technology is a strong candidate for a breakthrough in this area. The review has discussed recent successful attempts to reproduce synaptic plasticity and implement in-memory/in-sensor computations. Together with SNNs and the theory of the high-dimensional brain, the latter can produce novel approaches to neuromorphic computing. Then, SNNs can diverge from the development of ANNs and build their niche, cognitive, or reflective computations. The energetic efficiency and computational speed of future devices will be significantly improved. In turn, it may allow for overcoming the heat and memory walls that the current CMOS technology is facing.

## Author Contributions

All authors listed have made a substantial, direct, and intellectual contribution to the work, and approved it for publication.

## Conflict of Interest

The authors declare that the research was conducted in the absence of any commercial or financial relationships that could be construed as a potential conflict of interest.

## Publisher’s Note

All claims expressed in this article are solely those of the authors and do not necessarily represent those of their affiliated organizations, or those of the publisher, the editors and the reviewers. Any product that may be evaluated in this article, or claim that may be made by its manufacturer, is not guaranteed or endorsed by the publisher.
